# Lifestyle change in the cancer setting using ‘the teachable moment’: protocol for a proof-of-concept pilot in a urology service

**DOI:** 10.1186/s40814-016-0102-y

**Published:** 2016-10-21

**Authors:** Alyssa Sara Lee, Gozde Ozakinci, Steve Leung, Gerry Humphris, Hannah Dale, Neil Hamlet

**Affiliations:** 1 Department of Urology, NHS Fife, Kirkcaldy, UK; 2 School of Medicine, University of St Andrews, Fife, St Andrews, Scotland KY16 9TF UK; 3 Department of Psychology, NHS Fife, Kirkcaldy, UK; 4 Department of Public Health, NHS Fife, Kirkcaldy, UK

**Keywords:** Lifestyle change, Men, Cancer, Teachable moment, Behaviour change, Protocol, Activity, Diet, Alcohol, Smoking

## Abstract

**Background:**

Previous research has shown diagnosis or screening for cancer may be a ‘teachable moment’ for prevention through lifestyle change. Previous trials have been successful but have been delivered via national programmes targeting patients being screened for colorectal cancer. This manuscript reports the protocol for a proof-of-concept study to assess the feasibility and acceptability of a lifestyle change service targeting men suspected or diagnosed with cancer of the prostate in a secondary care cancer service within the UK.

**Methods:**

Lifestyle change will be promoted through integration of a lifestyle change service in a urology department in one NHS Board. The service is delivered by a Health Psychologist and uses motivational interviewing and behavioural change techniques to motivate and support patients to consider and address topics such as increasing physical activity and a healthy diet, smoking cessation, alcohol reduction and weight loss. A service evaluation will assess feasibility and acceptability via a patient experience survey, a survey exploring staff knowledge, attitudes and practice, pre- and post-intervention lifestyle behaviour survey and an audit of routine patient database.

**Discussion:**

This pilot will assess the viability of using cancer testing and diagnosis as a teachable moment for lifestyle change in a unique population (i.e. men with suspected cancer of the prostate). If successful, this approach offers potential for preventative services to enhance routine and person-centred clinical cancer care provided within secondary care settings.

**Electronic supplementary material:**

The online version of this article (doi:10.1186/s40814-016-0102-y) contains supplementary material, which is available to authorized users.

## Background

Research suggests that up to four in ten malignant tumours could be prevented through lifestyle and other environmental changes [1]. Globally, if these preventive approaches could be introduced, this would represent a major reduction in disease burden, improve population health, and reduce health services costs [2]. As part of the varied intervention approaches to tackling this issue, lifestyle change programmes situated within secondary care cancer services are an important consideration.

Previous research has shown that diagnosis or screening for cancer may be a ‘teachable moment’ for prevention. Although conceptualised in different ways in the literature, teachable moments are considered to be opportunities or contexts/events representing an increased desire, willingness or capacity for change in patients [3, 4]. Hospitalisations (or interactions with hospital settings) and/or health conditions that are potentially ‘life-threatening’ are those most associated with spontaneous lifestyle change efforts among patients [3].

Cancer of the prostate (CAP) is the most common cancer among men in the United Kingdom (UK), accounting for 25 % of all new cancers in males [5]. In 2012, there were 43,436 new cases of prostate cancer. The incidence of prostate cancer has increased since the late 1970s due in part to improved detection of the disease through prostate-specific antigen (PSA) testing. Such a test and/or a positive diagnosis may offer a so-called teachable moment for lifestyle change.

Lifestyle change is particularly relevant to men with or undergoing investigations for prostate cancer. The World Cancer Research Fund [6] recently published strong evidence for increased risk for developing advanced cancer of the prostate who are overweight/obese. Key health behaviours, which are amenable to change via lifestyle interventions (e.g. changing diet, physical activity, smoking and alcohol consumption), contribute greatly to obesity, other serious health conditions and shortened lifespan. General population tend to have poor levels of awareness and understanding of the relevant contribution of lifestyle in cancer risk [7, 8]. It is possible that knowledge regarding the importance of prevention through lifestyle change is particularly low among men seeking input from secondary care for suspected cancer compared to women.

Previous research has found that many men being tested for suspected cancer of the prostate display relevant risk factors [9, 10]. Lifestyle change can improve outcomes in this group [11, 12], but significant challenges remain. Men often experience barriers to accessing behavioural change advice and support [13]. Older males (especially those with lower educational status) are less likely to spontaneously change and/or proactively seek lifestyle support in relation to a cancer screen or a cancer diagnosis [13]. Furthermore, Larsen et al. [14] and Anderson et al. [15] argue that in the course of interactions with the health service, and in particular when a negative result is given to a patient being tested for a serious health condition, the absence of lifestyle change advice and support could be seen as endorsing current behaviour. The so-called health certificate effect may negate any heightened experience of risk or vulnerability arising from the context. Hence, the opportunity presented by a cancer test or diagnosis for developing patients’ motivation for change may be squandered (see work on colorectal cancer conducted by Stead et al. [8] and further studies below).

Previous research utilising the teachable moment concept has been successfully conducted in a cancer screening settings [16]. Most studies of this nature in the UK have been conducted in colorectal cancer possibly because of the centrally coordinated national screening programme [17–20]. Craigie and colleagues [17] found significant weight loss over a 12-month period, among men and women, undergoing three face-to-face consultations and monthly phone calls implementing a teachable moment intervention. This intervention consisted of colorectal cancer screening, motivational interviewing and behavioural change strategies targeting diet and physical activity. Other relevant clinical indicators also improved, and there was evidence of high feasibility and acceptability (e.g. high recruitment and retention rate, low per-patient intervention cost and proportionate uptake from across a range of socio-demographic groups). Despite this success, there are translational issues into existing secondary care cancer settings. Suggested reasons for poor uptake include lack of clinician and service commissioner preparedness for introducing the lifestyle advice in routine testing and treatment settings within secondary care [15].

Current UK and European guidelines do not advocate screening for CAP. The pros and cons of this are outwith the scope of this article and are discussed here [21]. However, similar to the successful work carried out in relation to the UK national colorectal cancer screening programme, a biopsy for suspected CAP or a diagnosis could potentially be utilised as a teachable moment for lifestyle change in secondary care cancer settings. To date, little is known about the potential feasibility and acceptability of this approach in these settings. If successful this approach has wide applicability across cancer services more broadly.

### Aims and objectives

The aim of the current study is to assess the feasibility and acceptability of a lifestyle change service targeting men suspected or diagnosed with CAP within a secondary care cancer service (urology) in the UK. The National Health Service (NHS) Urology Service offers diagnostics and treatment for men suspected with or diagnosed with prostate cancer. Diagnostics involve imaging and invasive procedures. Treatments options are varied but can involve major surgery, radiotherapy, chemotherapy or hormone therapy.

The lifestyle change service encompasses the following elements: it is integrated into existing participant treatment pathways (i.e. treated as a routine offer in the context of diagnostics/treatment); with access to patient information (exchanged via clinical systems, notes and through relationships with consultants and clinical nurse specialists (CNS)); assessment of lifestyle behaviours is conducted and normalised for patients; all potentially eligible patients (see criteria below) are invited at a specific moment to receive at least one face-to-face, one-to-one lifestyle consultation with a Health Psychologist (AL) who provides subsequent appointments as required. Finally, liaison with secondary and primary care colleagues is built into the service to enhance behavioural change and/or support a more holistic cancer care service. The development of the lifestyle change service and the associated evaluation is a proof-of-concept pilot. In other words, it is designed to identify whether a lifestyle change service, utilising teachable moments, can be integrated into routine cancer services for men with suspected, or diagnosed with, cancer of the prostate and has the potential for effecting positive change in this group and within the service. The specific objectives for this evaluation are to describe, assess and report:

1. Acceptability indicators
  - Uptake to the service from men with a negative cancer screen
  - Uptake to the service from men with a positive cancer screen
  - Difference in uptake using socio-demographic and clinical characteristics of patients
  - Consent for service evaluation
  - Patient experience and satisfaction with the service

2. Feasibility indicators
  - Number and duration of appointment(s) with a Health Psychologist
  - Number and type of behaviours discussed
  - Number of lifestyle change goals set
  - Length of time taken to compose letters
  - Changes to health behaviours made as a result of the consultation
  - Clinical staff knowledge, attitudes and practices regarding lifestyle change practice

## Methods/design

### Setting

The setting is a urology service within a Scottish secondary care site in one NHS Board. The service provides diagnostics and treatment for benign and malignant urological disease to a population of 380,000. In this NHS Board, 250 new prostate cancer diagnoses were made following approximately 500 prostate biopsies in 2014 [22].

The procedures for patients suspected of cancer of the prostate in this NHS Board are as follows: usually, patients undergo PSA testing and/or report relevant symptoms to their general practitioner within a primary care setting. If a specialist opinion is required, the patient is referred to the urology service in the secondary care setting. Specialist diagnostic investigations usually involve a transrectal ultrasound (TRUS) guided biopsy of the prostate or magnetic resonance imaging (MRI) of the prostate. The TRUS biopsy procedure is carried out by Consultant Urologists. The prostate cancer CNS co-ordinate appropriate adjunct investigations and provide patient support throughout the cancer pathway.

### Design

The study is a proof-of-concept pilot. The service evaluation will use a mixture of methods to assess feasibility and acceptability of a teachable moment intervention for lifestyle change within this novel setting. This includes a patient experience survey, a staff experience survey exploring knowledge, attitudes and practice, a lifestyle survey (pre- and post-intervention patient lifestyle behaviour change survey) and an audit of routine patient data collected for service feasibility and delivery outcomes.

There is no control group. This is a service evaluation rather than a pilot trial.

### Participants

All male patients referred to the urology service with a suspicion of cancer of the prostate, who undergo a TRUS biopsy of prostate, will be invited to participate in the intervention and service evaluation. Men who have been diagnosed with low-grade cancer of the prostate and who have made a treatment decision to proceed on an active surveillance protocol will also be invited to participate in the service and evaluation. Active surveillance is the postponement of immediate treatment, with definitive treatment provided if there is evidence that the patient is at increased risk for disease progression.

### Intervention

#### Procedures for the intervention

All patients will be invited to receive at least one face-to-face, lifestyle consultation with a Health Psychologist. Consultations will run at the hospital clinic and are expected to last between 30 and 90 min. The intervention will be individualised for all patients who come through the service but will include some key features (see details below). Patients will be advised that they can bring a spouse or other supporter/family member.

Where possible, appointments will run immediately after seeing the CNS for TRUS biopsy results (2 weeks following biopsy), if patients are to be given a negative result. The CNS will ask patients if they are willing to participate in the new service. Patients will already have been ‘primed’ to expect this through a letter which explains the importance of their lifestyle as part of their urology care. A pre-procedure lifestyle survey is also included (see procedure for evaluation below), which all patients will be invited to complete before attending the biopsy appointment. When the Health Psychologist is unavailable on the day of the results clinic, a follow-up telephone call will be made to arrange an appointment.

Men who receive the active surveillance protocol will be sent a hospital letter advising them to contact the service to make an appointment for a lifestyle consultation. Patients who pro-actively contact the service will be followed up to arrange an appointment with the Health Psychologist.

#### Consultation style and content

Consultations will start with rapport building plus a discussion of the role of the Health Psychologist as part of the wider urology team and will use a combination of approaches to discuss and prompt lifestyle change efforts. This will include using motivational interviewing (MI) [23] and behavioural change techniques (BCTs), taken from the taxonomy of BCTs [23]. The theoretical underpinnings of MI and BCTs are well understood [23, 24].

MI is defined as ‘a directive, client-centered counseling style for eliciting behavior change by helping clients to explore and resolve ambivalence’ ([24] (para. 3)). Although the focus of the consultation will be patient-centred (e.g. if they raise a concern about their weight) in keeping with the goal-directed nature of MI, it will also be monitored by the Health Psychologist who will seek to use the consultation to explore and resolve ambivalence about making relevant lifestyle changes. In keeping with MI, resistance will be met with reflective statements and emphasis on personal agency of the patient, especially regarding uncertainty and ambivalence about changing. Difficult and competing emotions associated with making a change will be normalised and empathy expressed with the patient experience. The importance of quality of life and long-term goals for health will be raised by the Health Psychologist to match patients’ concerns that are raised (e.g. positive health is promoted and not just a lack of illness). Patients will be advised that looking holistically at all aspects of their lifestyle is important for the team to help identify areas where changes could be made to improve their own personal outcomes (e.g. quality of life) in addition to improving health outcomes. Some examples will be used, such as stopping smoking and increasing exercise. Patients will be explicitly advised that the consultation will follow their agenda for lifestyle topics focusing on what patients noticed from their completed lifestyle survey and/or what they had heard about the role of lifestyle in cancer prevention and treatment. Efforts will be made to socialise patients to a collaborative approach as distinct from their usual expectations of secondary care appointments which follow a more traditional medical model. This will be achieved through agreeing goals together, asking patients for their views and problem-solving before devising detailed behaviour change suggestions are made. Sometimes there will be an explicit, open discussion to contrast deliberately this approach with that of previous medical encounters that the patient may have experienced. Emphasis will be placed on participants as the judge of what is right for them in deciding to make a lifestyle change attempt.

Therefore, the conversational style, or approach, to the consultation will follow a MI framework. BCTs will be drawn upon within this style to bring further evidence-based intervention approaches to the consultation. Discussions will include some, or all, of the following BCTs, depending on the appropriate use of the BCT in the context of the patients’ presentation: evidence-based information provision on recommendations for cancer prevention (or overall good health) when the participant has indicated they want it or are open to this information. Where indicated, patients will be given or shown credible written information (e.g. European code on Cancer Prevention, World Cancer Research Fund information). Individualised information will be used by highlighting or affirming patients’ existing knowledge regarding positive lifestyle behaviours. Past experiences of successful behavioural change will be highlighted and affirmed. Efforts will be made to identify what made those changes successful to raise patient self-efficacy for new change attempts. Furthermore, patients will be asked what outcomes they experienced from changing (e.g. felt fitter, better, received compliments) or in the absence of such experience what they imagined they would experience if they made a change. In some cases, patients will make a commitment to make one or more behavioural changes. This could occur at the first appointment (where the assessment is conducted and the intervention begins or during later sessions if warranted). Usually, change goals will be developed using a written booklet (materials that had been previously developed by a Health Psychologist for NHS Fife, such as healthy eating action plans and physical activity plans). Patients will be expected to set between one and four specific goals (e.g. reduce alcohol consumption by having one to two alcohol free days per week, lose 20 lb in 3 months, take a brisk walk twice per week for at least 1 hour). Patients will be encouraged to plan when, and where, and how often each week, they will perform the behaviour (e.g. walking at 3 pm on Monday and Wednesday). Some patients will be encouraged to set a reward for effort or progress towards their goal or to access relevant social support. Patients will be encouraged to monitor behaviour and/or outcome (e.g. weight) closely and/or to complete if-then (implementation intention [25]) plans so they have a backup to their initial behavioural change plan. This is not an exhaustive list from the BCT taxonomy [23], and the full range of cognitive and behavioural techniques are used according to the different consultations and needs of patients. Examples of technique usage are given in Additional file 1. Lastly, communication with primary and secondary care colleagues will be conducted to inform them of intervention with the aim of enabling further support for change when patients attend other NHS appointments.

#### Number and delivery mode for consultation sessions

Up to 12 consultation sessions will be available for patients by phone or face-to-face appointments in the hospital site. However, in practice, most participants will be likely to receive fewer consultations than this. Follow-up sessions will be negotiated with the patient depending on a number of factors including:

- *Patient preference* (e.g. contact by telephone due to location of hospital clinic or prefer no follow-up if not wishing to pursue lifestyle change goals)
- *Feedback from patients* regarding goal attainment/difficulty as patients’ progressed through behavioural change attempts (e.g. patients not achieving goals or feeling demotivated could be brought back in for further face-to-face consultation)
- *Clinical expertise* of the Health Psychologist (e.g. identifying patients who likely require a greater level of input)

### Service evaluation

#### Procedures

Data collection procedures are dependent on the type of data being extracted for evaluation. Table 1 below summarises procedures for collecting, analysing, and reporting each indicator for the service evaluation. All information is entered into routine databases that are used for service delivery. Data extraction is via an audit of this information for the duration of the service delivery period (at the end of proof-of-concept pilot).

**Table 1:** Summary of the service evaluation procedures

Data collection indicator	Method of data collection	Timing of data collection	Planned analysis and reporting
Acceptability indicators			
(i) Uptake to the service from men with a negative cancer screen	Number of men receiving a TRUS biopsy who are invited to take part in the service via CNS face-to-face contact is logged via a routine audit database. Those who access at least one appointment for lifestyle change will be used as positive for uptake^a^.	Ongoing throughout service delivery.	Descriptive analysis using Excel. Reported as proportion using numerical units and percentages.
(ii) Uptake to service from men with a positive cancer screen	Number of men diagnosed with cancer of the prostate in the previous calendar year (choosing an active surveillance protocol for cancer management) who are invited to take part in the service via patient letter is logged via a routine audit database. Those who access at least one appointment for lifestyle change will be used as positive for uptake^a^.	Ongoing throughout service delivery.	Descriptive analysis using Excel. Reported as proportion using frequency counts with percentages.
(iii) Difference in uptake using socio-demographic and clinical characteristics of patients	Age (at invite for appointment), deprivation category (from postcode), named consultant and type of patient (cancer/no cancer diagnosis) are taken from patient information summary and logged via a routine audit database^a^.	Ongoing throughout service delivery.	Logistic regression using STATA to use socio-demographic and clinical characteristics as predictors of accessing the service. Reported using odds ratios and 95 % confidence intervals.
(iv) Consent for service evaluation	Patients are asked to provide consent to be involved in the service evaluation during their first lifestyle appointment. Numbers of men consenting logged via a routine audit database^a^.	Ongoing throughout service delivery.	Descriptive analysis using Excel. Reported as proportion using frequency counts with percentages.
(v) Patient experience and satisfaction with the service	All patients are sent a short self-completion postal survey to gather information on their experiences of the service (patient experience survey). Data collection is via a postal questionnaire, which is sent to them along with a covering letter, and is returned via a freepost envelope. Information is entered into a separate database. The survey asks for a response (i.e. tick box and return of survey) even if the patient decides not to participate. More detailed information below.	Patients are sent an anonymous questionnaire 2–4 weeks after their first appointment.	Descriptive analysis using Excel. Reported as proportion using frequency counts with percentages. Satisfaction data reported as mean (s.d.). Open-ended comments analysed thematically (with counts) and a selection reported as direct quotations.
Feasibility indicators			
(i) Number and duration of appointment(s)	Each appointment date, length and type (face-to-face or telephone) for each patient is logged via a routine audit database^a^.	Ongoing throughout service delivery.	Descriptive analysis using Excel. Reported as median number, length with IQR and type of appointment using frequency counts with percentages.
(ii) Number and type of behaviours discussed	A record of the number of health issues and behaviours (amenable to change) that are discussed with patients is logged via a routine audit database. These are split into primary and secondary issues/behaviour (e.g. if weight loss is the main area of discussion this would be coded as *primary* with diet, physical activity and alcohol use coded as *secondary* areas).	Ongoing throughout service delivery.	Descriptive analysis using Excel. Reported as median total number (and for each behaviour) using frequency counts with percentages.
(iii) Number of lifestyle change goals set	A record of the number and type of goals set by patients is logged via a routine audit database^a^.	Ongoing throughout service delivery.	Descriptive analysis using Excel. Reported using frequency counts with percentages.
(iv) Length of time taken to compose letters	Time taken to compose patient letters is logged for each patient via a routine audit database^a^.	Ongoing throughout service delivery.	Descriptive analysis using Excel. Reported as median with IQR.
(v) Changes to health behaviours	All patients taking part in the TRUS biopsy procedure are sent a short self-completion lifestyle survey (lifestyle survey as par covering several items (see detailed information below)). This is sent to them along with a covering letter describing the TRUS procedure (routine practice). Patients return this survey in person to the CNS at the first appointment who passes this information on the Health Psychologist. In some cases, patients may complete the survey during the first appointment (if not otherwise completed). Active surveillance patients receive the lifestyle survey via a letter confirming their appointment for a lifestyle consultation. They are asked to bring the survey with them to their appointment. In some cases, patients may complete the survey during the first appointment (if not otherwise completed). For repeat data collection, a postal survey is sent out to patients. If patients have failed to respond within 2–4 weeks, up to 3 phone calls are made to remind the patient about completion of the survey. The repeat survey can be completed over the phone during this call if preferred. Patients are asked to provide consent to be involved in the service evaluation during their first lifestyle appointment. Information from the lifestyle survey is entered into a separate database. More detailed information below.	For TRUS patients, the lifestyle survey is completed prior to attending their biopsy appointment. For active surveillance patients, the lifestyle survey is completed prior to their first consultation appointment. A repeat survey is completed approximately 3 months following their first appointment to assess any pre-post changes.	Descriptive analysis using Excel. Using a coding system patient lifestyle change from pre-post is coded as *worse*, *no change*, *moderate* or *substantial change* in their primary and secondary health behaviour areas (those discussed during the consultation). Reported using frequency counts with percentages. See more detailed information below.
(vi) Clinical staff knowledge, attitudes and practices regarding lifestyle change practice	Questionnaire sent by email via to all consultant urologists and clinical nurse specialists in the Department. Data collection via online Survey Monkey. Information is entered into a separate database. Staff consent to be involved in the survey. Consent is implicit and confirmed via their participation in the survey. Information is collected anonymously.	Sent approximately half way into proof-of-concept pilot. Reminder email sent 2–4 weeks later requesting response.	Descriptive analysis using Excel. Knowledge, attitude and practice information reported via mean (s.d.) for each item. Open-ended comments analysed thematically (with counts) and a selection reported as direct quotations.

^a^Consent not required due to routine audit of non-identifiable patient data

#### Patient experience survey

As described in Table 1, consenting patients who receive at least one lifestyle consultation will be sent an anonymous patient experience survey. This will collect information through eight items including: *age* (patients mark this using an *X* on a ruled line), their *memory of taking part in the consultation* (yes/no/not sure), their *satisfaction with the service* (5-point Likert scale: very satisfied/satisfied/neither satisfied or unsatisfied/unsatisfied/very unsatisfied), their *perception of benefit* (yes/no/not sure) and *would they recommend to other men in a similar position* (yes/no/not sure). Patients will be also asked open-ended questions about what they most liked/disliked about aspects of service. Space for written comments is also provided with each question. The survey is sent out 2–4 weeks after the first appointment and returned via a prepaid envelope. Analysis is described in Table 1.

#### Staff survey

As described in Table 1 consenting staff from the Department of Urology will be sent the knowledge, attitudes and practice survey by email. This collects 18 data items including *workplace demographics* (4 items)—(i) *clinical role* (consultant/CNS); (ii) *level of experience* (less than 5 years/5–10 years/10 years or more); (iii) *working hours* (full/part-time) and (iv) *gender* (male/female). *Knowledge*, *attitude and practice* (14 items) were created by the research team to assess agreement with statements about lifestyle change. Staff will be first advised:

Please tell us how much you agree with the following statements about lifestyle change. By lifestyle change we mean anything that your patients can do to promote their own health and wellbeing. This could include general changes that most health professionals might recommend for living a healthy lifestyle, or specific changes, that could help patients manage symptoms or improve the effectiveness of the treatment you recommend for them. Some examples: cutting down on alcohol or caffeine, stopping smoking, taking more exercise, improving their diet, taking medication as prescribed and taking steps to managing stress etc.

The choice of items was guided by constructs from Theory Domains Framework (TDF) [26], which is a theoretical framework developed through expert consensus and factor analysis. It uses psychological theory to explain the facilitators and barriers of health professional practice (largely focused on provision of lifestyle change advice and support). TDF has previously been used to assess implementation of lifestyle change practice across a number of clinical areas [27].

Following iterative review with the research team, 14 items were developed to assess the following facets: knowledge; skills; social/professional role and identity; beliefs about capabilities; optimism; beliefs about consequences; reinforcement; intentions; goals; memory, attention and decision processes; environmental context and resources; social influences; emotion and behavioural regulation. The item wording is available in Additional file 2. All knowledge, attitude and practice items were rated using a 7-point Likert scale from *strongly disagree* to *strongly agree*. Analysis is described in Table 1.

#### Lifestyle survey

As described in Table 1, all patients will complete a short lifestyle survey prior to receiving the intervention and at around 3 months follow-up. This will collect information about 15 items focussing on the following behaviours:

- *Smoking* (2 items): Patients will be asked ‘Do you smoke tobacco?’ and respond as current smoker/ex-smoker/no (i.e. never smoker; current smokers write down number smoked per day)
- *Alcohol* (3 items): Patients will be asked ‘Do you drink alcohol?’ and respond as most weeks/occasionally/no (i.e. non-drinker; current drinkers to write down the highest number of units consumed per day and units consumed per week)
- *Fruit and vegetable consumption* (2 items): Patients will be asked how many portions of fruit and vegetables they consume in separate questions and report number of each consumed per day; for analysis, figures are added for total consumption
- *Height* (1 item) and *weight* (1 item): Weight is a relevant outcome, and this also allows for calculating body mass index. Patients will be also asked when was the last time they weighed themselves/were weighed (1 item with possible responses: within the last month/within the last 6 months/more than 6 months ago/don’t know)
- *Caffeine intake* (1 item): Patients will write down the number of caffeinated products consumed by viewing a list of tea/coffee/cans of cola/energy drink(s)/bars of chocolate (plain/milk). The total daily caffeine intake will be calculated by the following using data from the conversion information cited in Knight et al. [26] (e.g. number of tea mugs × 60 mg and then added for together for each product to get a total mg of caffeine)
- *Physical activity* (2 items): Patients will be asked separately ‘On how many days have you been physically active for a total of 30 min or more in the PAST WEEK?’ and ‘On how many days did you do physical activity that strengthened your muscles in the PAST WEEK?’. There is an explanation of what intensity is required (noticeable increase breathing/slow walking does not count) and that 10-min bout of activity is the minimum needed for inclusion
- *Stress management* (1 item): Patients will be asked ‘What do you do to help you manage STRESS?’ and respond by ticking agreement with a list of common techniques (e.g. say no to things/take control of difficult things/express your feelings/accept things you can’t change/make time to relax and enjoy life) with space for other (give details) or not relevant to me
- *Worthwhile activities* (1 item): Patients will be asked ‘How much do you take part in activities in your life that are worthwhile TO YOU?’ and will respond via a 5-point Likert scale: none of the time/a little of the time/some of the time/most of the time/all of the time.

The lifestyle survey was developed as a pragmatic method for assessing behaviours quickly. Questions are worded to be as clear as possible and using written and visual information to improve accuracy of calculations (e.g. providing a visual of typical drinks and unit calculations for each next to the alcohol questions).

The survey was developed iteratively (first, with the research team and, second, through a pilot with eight TRUS biopsy patients routinely attended the urology clinic, who were not receiving the intervention described above). Patients reviewed earlier drafts of questions by completing the survey. Patients were asked to give feedback, while they waited to see the CNS. Time for completion was recorded for each patient, and patients were asked verbally to explain questions they were not sure about or had trouble with, questions they did not want to answer and how accurate and truthful they felt their responses had been, as well as any other comments.

#### Analysis

As described in Table 1, all data from the lifestyle survey will be entered into a spreadsheet as surveys are returned. To assess change from pre-post across these diverse behaviours, each item will be coded as *worse*, *no change*, *moderate or substantial change.* The criteria for coding and rationale are explained fully in Table 2 with details in Additional file 3. Criteria are focused on guidelines from the UK on optimal performance of health behaviours such as a reduction of alcohol units to within weekly national guidelines for males (where exceeding guidance prior to the consultation) was considered a substantial change.

**Table 2:** Summary of the coding system for the lifestyle change outcomes

Behaviour/outcome	Units of measurement	Comment	Worse	No change	Moderate change	Substantial change	Evidence for choices
Smoking status	Current smoker, ex-smoker, non-smoker		Ex- and non-smoker became a smoker	No change to current smokers smoking status	n/a	From initial smoker status becomes ex-smoker at follow-up	
Smoking amount	How many smoked per day (including cigarettes, roll-ups, cigars, pipes)?	Only relevant to current smokers	Increased number smoked per day at follow-up	No change to number smoked per day at follow-up	≥50 % reduction to number smoked per day at follow-up	From initial smoker status becomes ex-smoker at follow-up	NICE (2013) guidance on harm reduction for tobacco^a^
Alcohol status	Drink alcohol? Most weeks, Occasion-ally, No		Non-drinker became occasional/most weeks drinker OR Occasional drinker became most weeks drinker	No change to current drinking status	Occasional drinker becomes non-drinker OR	Most weeks drinker becomes occasional or non-drinker	Recommendation from the European Code Against Cancer^b^
Alcohol units	Most units drunk per day	Only relevant to current drinkers	Increased number of units per day at follow-up	No change to units drunk	Some reduction—≤50 % in units drunk per day at follow-up	Drinker exceeding daily limit at baseline is under daily limit at follow-up (i.e. ≤4 units per day) OR ≥50 % reduction in units drunk per day at follow-up	Alcohol unit guidelines from UK Government^c^
Alcohol units	Total units drunk per week	Only relevant to current drinkers	Increased number of units per day at follow-up	No change to units drunk	Some reduction—≤ 50 % in units drunk per week at follow-up	Drinker exceeding weekly limit at baseline is under weekly limit at follow-up (i.e. ≤21 units per week) OR ≥50 % reduction in units drunk per week at follow-up	Alcohol unit guidelines from UK Government^c^
Weight loss	% weight loss (in kg)	All men—but particularly relevant to overweight/obese men	Increase in weight	No change to weight	Some reduction in weight (<5 % at follow-up)	Reduction in weight is ≥5 % at follow-up	Research showing a 5 % reduction in weight leads to health benefit^d^
Body mass index	BMI (i.e. weight (kg)/height (m^2^))	All men—but particularly relevant to overweight/obese men	Increase in BMI	No change to BMI	Reduction in BMI maps onto a <5 % reduction in weight at follow-up	Change in BMI category (i.e. from Obese to overweight or from overweight to healthy weight range) OR Reduction in BMI maps onto a ≥5 % reduction in weight at follow-up	Research showing a 5 % reduction in weight leads to health benefit^d^
Physical activity (cardiovascular)	Number of days active for at least 30 min (≥moderate intensity)	All men	Any reduction in number of active days (CV)	No change to number of active days (MS)	Increase in number of active days (CV) by 1	Increase in number of active days (CV) by >1 day OR Achieves weekly physical activity (CV) guidelines day at follow-up (only those not achieving them at baseline)	UK physical activity guidelines for adults^e^
Physical activity (muscle strengthening)	Number of days any muscle strengthening activity	All men	Any reduction in number of active days (MS)	No change to number of active days (MS)	Increase in number of active days (MS) by 1	Increase in number of active days (MS) by >1 day OR Achieves weekly physical activity (MS) guidelines day at follow-up (only those not achieving them at baseline)	UK physical activity guidelines for adults^e^
Fruit and vegetable intake	Total daily portions of fruit and vegetables	All men	Any reduction in number of fruits and vegetables eaten	No change to number of fruits and vegetables eaten	Increase in portions of fruits and vegetable by 1 per day	Increase in portions of fruits and vegetable by >1 per day OR Achieves five portions FV per day at follow-up (only those not achieving 5 a day at baseline)	Current NHS guidance plus research evidence^f^

^a^According to NICE [32], it is currently unknown how great the health benefits of smoking reduction are (by substituting some cigarettes with licensed nicotine-containing products) compared to stopping smoking. This is a research area they recommended. However, it is also noted that people from routine and manual groups are more likely to cut down first, rather than stop ‘abruptly’, and intervention studies showed a positive effect where the primary outcome was to help people cut down prior to stopping smoking (mainly cognitive behavioural therapy and counselling)

^b^According to the European code against cancer [34], if you drink alcohol of any type, limit your intake. Not drinking alcohol is better for cancer prevention

^c^The Royal College of Physicians evidence base for alcohol guidelines [35]

^d^The following studies have shown significant decreases in triglycerides, waist circumference, glucose, insulin and blood pressure following a minimum 5 % weight-loss [36, 37]

^e^Recommendations from the four Chief Medical Officer’s in the UK [38] include 2½ h CV per week (i.e. 5 * 30 min). Adults should also undertake physical activity to improve muscle strength on at least 2 days a week. There are also guidelines for older adults (including balance exercises); this was not included in the lifestyle survey. Sedentary time was not included either despite being part of the new guidelines

^f^Wang et al. [39] showed a threshold for all-cause mortality from fruits and vegetable consumption of five per day

When reporting change in lifestyle behaviours, each patient’s own primary (most important or topic encompassing most time during the consultation) and secondary health behaviour topics will be reported only (i.e. across the range of possible health topics, we will report the number and proportion showing change/no change in an area discussed during contact with the service). This will provide information on potential impact of the teachable moment intervention on lifestyle behaviours across the spectrum discussed.

#### Audit of routine patient database

A routine data collection database was developed to be used for service delivery. However, indicators that will be captured here include the following items that are reported on below (required to assess feasibility and acceptability of the service):

i) Of each patient who was invited whether they accepted and received an appointment with the Health Psychologist
ii) Each patient’s socio-demographic and clinical characteristics
iii) Whether consent for service evaluation was provided
iv) The total number of, and duration of, appointment(s) with the Health Psychologist
v) The number and type of behaviours discussed with the Health Psychologist
vi) The number and nature of lifestyle change goals set
vii) The length of time taken to compose letters regarding appointments

Table 1 below summarises procedures for collecting, analysing and reporting each indicator for the service evaluation. Data extraction was via audit process and service evaluation for the duration of the service delivery period (at the end of proof-of-concept pilot).

## Discussion

This work describes the initiative to introduce lifestyle consultations for behaviour change that take advantage of cancer testing and/or diagnosis as a teachable moment for change. As a proof-of-concept pilot, the scope of this work is to assess the feasibility and accessibility of such a development in a busy urology service (providing secondary care cancer services).

In this pilot study, a Health Psychologist is used to conduct lifestyle consultations, and the patient experience data and lifestyle survey audit will provide information regarding the feasibility of this approach. However, in previous work using ‘teachable moments’ in a trial within colorectal cancer screening ‘trained lifestyle counsellors’ were used [17]. The extent to which existing clinical staff within secondary care cancer settings (e.g. consultant doctors and CNS) could be used is an important area for exploration. However, previous translational evidence has shown such staff often struggle with elements of implementing lifestyle change with patients (particularly using MI) [28]. Our study uses a staff survey with existing clinical staff to tease out whether knowledge, attitudes and practices might make integrating lifestyle change into routine health service conversations more or less feasible for these professionals.

The importance of testing the feasibility and acceptability of this approach to integrating lifestyle change into practice should not be underestimated. Numerous authors and public agencies have recognised the problems in translating the results of high-quality clinical trials of preventative approaches into practice [29, 30]. As described by Basch et al. (as cited in Glasgow and colleagues [29]), ‘a program cannot be effective if it is not implemented’ (p. 1256). This type of proof-of-concept testing should, therefore, be welcome and, if successful, should offer service managers and clinicians reassurance that such services add rather than take away from the routine and person-centred clinical cancer care they provide. It should also be recognised that any lifestyle change discussion following a consultation regarding a cancer diagnosis needs to be dealt with required care to take the emotional state of the patient into account and avoid any possibility for implying blame in the patient’s health status.

Indeed, this development should not be seen in isolation, but rather in the context of the national public policy framework operating within Scotland, which places early detection, and the prevention of cancer, as well as timely and high-quality treatment and post-treatment as central to the agenda for service innovation and improvement [31, 32]. There are also numerous other cancer testing, screening and treatment settings in which lifestyle change represents an important part of improving patient outcomes (both from a preventative and treatment perspective). Overall, if successful, this work could present one viable option for integrating preventative approaches into such services more widely. We believe that new service inputs into the health care system can be introduced as pragmatic, measurable elements which are certainly amenable to evaluation and thereby contribute to expanding the evidence base. However, in order to achieve wider implementation, effectiveness of this service would need to be shown through a larger study with multiple Health Psychologists delivering the service and testing the intervention through a randomised trial.

## Additional files

Additional file 1: Examples of other behaviour change techniques used in the first and subsequent consultations*. (DOC 24 kb)
Additional file 2: Staff survey knowledge, attitudes and practice items (developed using Theory Domains Framework constructs) [33]. (DOC 24 kb)
Additional file 3: Coding criteria for lifestyle change outcomes (pre-post). (DOC 23 kb)

## Abbreviations

BCTs: Behaviour change techniques
CAP: Cancer of the prostate
CNS: Clinical nurse specialists
MI: Motivational interviewing
MRI: Magnetic resonance imaging
TDF: Theory Domains Framework
TRUS: Transrectal ultrasound
UK: United Kingdom
