# Supplementary material for: Lifestyle change in the cancer setting using ‘the teachable moment’: protocol for a proof-of-concept pilot in a urology service
Source: Pilot Feasibility Stud. 2016 Oct 21;2:65. doi: 10.1186/s40814-016-0102-y (PMC5154035; doi:10.1186/s40814-016-0102-y)
Supplement: Additional file 1: — Examples of other behaviour change techniques used in the first and subsequent consultations*. (DOC 24 kb) [file 40814_2016_102_MOESM1_ESM.doc]

**Additional file 1 – Examples of other behaviour change techniques used in the first and subsequent consultations***

- Encourage patient to use written prompts (e.g. keep their diet plan on the fridge)
- Instructions on how to perform the behaviour (e.g. use a pedometer to self-monitor step count then add 1000 steps or 10 minutes of brisk walking three times per week, reduce portion size)
- Reframing (e.g. not having gained weight despite holiday as positive progress, stuck to not smoking despite period of stress)
- Encouraging a realistic change for longer-term (e.g. highlight value of small amount of weight loss and change to eating plan rather than diet)
- Normalised difficult of change (e.g. weight loss is a learned skill with peaks and troughs)
- Discussions of small environmental changes key to success (e.g. using smaller plates, not buying biscuits, using a different shop which doesn’t promote unhealthy products, writing a shopping list)
- Information/reminders about other’s approval or other benefits of change (e.g. spouse or doctor or family desire to see them quit smoking, will allow them to get operation they need)
- Psychoeducation (e.g. about the importance of mood and attitude in maintaining motivation and effort or about the difficulty in establishing new habits as a rationale for introducing written plans or behavioural techniques)
- Highlight resilience factors in their favour (e.g. supportive family, financial security, previous experience of sport)
- Building on patient own existing behaviours (e.g. reduced drinking on golf days when driving extends to other days)

*Techniques are those described and discussed in the Behaviour Change Taxonomy
