# Supplementary material for: Lifestyle change in the cancer setting using ‘the teachable moment’: protocol for a proof-of-concept pilot in a urology service
Source: Pilot Feasibility Stud. 2016 Oct 21;2:65. doi: 10.1186/s40814-016-0102-y (PMC5154035; doi:10.1186/s40814-016-0102-y)
Supplement: Additional file 2: — Staff survey knowledge, attitudes and practice items (developed using Theory Domains Framework constructs) [33]. (DOC 24 kb) [file 40814_2016_102_MOESM2_ESM.doc]

**Additional file 2 – Staff Survey Knowledge, Attitudes and Practice Items (developed using Theory Domains Framework Constructs)**

1. I know what types of lifestyle changes I should recommended during consultations with my patients.

2. I have the skills to discuss lifestyle changes during consultations with my patients.

3. People in my position should discuss lifestyle changes during consultations with my patients.

4. I am confident I can discuss lifestyle changes during consultations with my patients.

5. Generally speaking, I am optimistic that things will turn out for the best.

6. If I discuss lifestyle changes during consultations with my patients it is more likely that they will take steps to change their behaviour.

7. In my workplace discussing lifestyle changes during consultations with my patients is rewarded.

8. I have every intention to discuss lifestyle changes during consultations with my patients.

9. I always plan how I am going to discuss lifestyle changes during consultations with my patients.

10. I often forget to discuss lifestyle change with my patients during consultations.

11. The number of patients I have in my clinic influences how much I discuss lifestyle changes during consultations with my patients.

12. The views/opinions/support of other staff affects how likely I am to discuss lifestyle changes during consultations with my patients.

13. Tackling lifestyle changes during consultations with my patients is emotionally challenging.

14. I am fully aware of how much I discuss lifestyle changes during consultations with my patients.
