# Supplementary material for: Lifestyle change in the cancer setting using ‘the teachable moment’: protocol for a proof-of-concept pilot in a urology service
Source: Pilot Feasibility Stud. 2016 Oct 21;2:65. doi: 10.1186/s40814-016-0102-y (PMC5154035; doi:10.1186/s40814-016-0102-y)
Supplement: Additional file 3: — Coding criteria for lifestyle change outcomes (pre-post). (DOC 23 kb) [file 40814_2016_102_MOESM3_ESM.doc]

**Additional file 3 – Coding criteria for lifestyle change outcomes (pre-post)**

As the patients seen by the Health psychologist may have worked on a variety of different health behaviour outcomes, and given there is not a uniform type of unit across these categories; it was agreed that the outcomes of change would be coded for summary.

The codes are as follows:

- ***Worse*** (i.e. increased alcohol consumption or reduced physical activity participation) at follow-up
- ***No change*** at follow-up
- ***Moderate*** change at follow-up (see Table 2)
- ***Substantial*** change at follow-up (see Table 2)

Each outcome will have values ascribed for what worse, no change, and moderate and substantial changes means. Only the following behaviours/outcomes will be included:

- Smoking
- Alcohol
- Physical activity
- Diet
- Weight loss
